# Immediate effects of yoga on anxiety, depression, and sleep in women with chronic pain in a rural community setting: a pilot feasibility study

**DOI:** 10.3389/fmed.2025.1671950

**Published:** 2025-11-03

**Authors:** Alexandro Andrade, Anderson D’Oliveira, Carina Jorge da Silveira Moreira, Stefania Mancone, Pierluigi Diotaiuti, Micheline Henrique Araujo da Luz Koerich

**Affiliations:** 1 Health and Sports Science Center - CEFID, Santa Catarina State University - UDESC, Florianopolis, Santa Catarina, Brazil; 2 Laboratory of Sports and Exercise Psychology - LAPE, Florianopolis, Santa Catarina, Brazil; 3 Department of Human Sciences, Society and Health, University of Cassino and Southern Lazio, Cassino, FR, Italy; 4 Department of Physiotherapy of the Center for Health and Sports Sciences - CEFID, State University of Santa Catarina - UDESC, Florianopolis, Santa Catarina, Brazil

**Keywords:** anxiety, depression, mental health, pain, sleep quality, yoga

## Abstract

**Objective:**

This randomized pilot study assessed the feasibility and acceptability of a remote yoga intervention and examined potential effects on pain, sleep and the psychological variables anxiety and depression.

**Method:**

The intervention for the experimental group consisted of Hatha Yoga sessions, guided by video, lasting 60 min, divided into three moments: stillness and awareness of breathing, practice of postures (asanas), and meditation. Participants randomized to the intervention group participated in guided group video practice sessions for 2 weeks in the community church hall. Participants randomized to the control group were instructed to maintain their normal daily routine. The feasibility measures evaluated were: acceptance rates, ability to complete the intervention, external interference from other physical activities, and adverse effects. Outcome measures (self-reported pain, anxiety, depression, and sleep quality) were assessed at baseline and post-intervention. The Wilcoxon test was used to analyze the differences between groups, and the Mann–Whitney U test to compare the control group and the different moments of the intervention group. The effect size was assessed using Cohen’s *d*.

**Results:**

In the comparisons between the groups, there were no significant differences in any of the outcomes. The group that performed the intervention presented a significant reduction in anxiety. In addition, improvements were observed in the intervention group in pain, depression, and sleep quality according to the mean values, with a small to moderate effect size. Regarding the feasibility measures, the study had excellent acceptance rates; all participants completed the intervention. There was no external interference from other physical activities of the groups, and no serious adverse effects were reported.

**Conclusion:**

The asynchronous Hatha Yoga intervention was feasible, well accepted, and safe to be applied remotely in a group of women with chronic pain, living in a rural community. Despite the short duration of the Hatha Yoga intervention, the data presented suggest that improvements in anxiety, pain, depression, and sleep quality were observed in women with chronic pain. Large-scale randomized controlled trials are suggested to evaluate the effects of this modality on pain-related and psychological outcomes.

## 1 Introduction

Chronic pain is defined as pain persisting for more than 3 months and is often associated with functional disability and emotional distress (1). This condition is highly prevalent among older adults (2, 3), negatively affecting the quality of life, mental health, and functional capacity of this population (4, 5). Evidence suggests that women are more likely than men to experience chronic pain, reporting greater intensities, frequencies, and durations of pain and depression (6).

Diotaiuti et al. (7) highlighted that attentional focus and cognitive orientation can significantly modulate the perception of pain, providing theoretical support for mind–body practices, such as yoga, in managing chronic pain.

In this sense, mind–body interventions, have been included as part of the therapeutic approach to chronic pain (8), combining physical postures (asana), breathing techniques (pranayama), and meditation (dyana) to promote physical and mental wellbeing (9). Focusing on deep controlled breathing, attention to physical comfort, redirecting attention, mindful practice, relaxation, and slow, gentle movements can produce better awareness of afferent (sensory) feedback, allowing for a more effective response to efferent (motor) commands, reducing muscle tension and spasms associated with pain (10).

Systematic reviews (11–13), have shown the effects of yoga in reducing pain and disability in people with chronic musculoskeletal pain. However, these studies did not explore the impact of this intervention modality on psychological outcomes and/or sleep disorders in this population. Studies carried out with healthy older people point out that yoga interventions improve outcomes such as depression, mental health, vitality, sleep quality, and quality of life (14). Yoga seems to promote healthy psychological responses, indicating its potential as a strategy for emotion regulation.

In most clinical studies, yoga sessions are led in person by an instructor (15). However, access to this intervention format can be a major barrier for residents of rural areas, due to the unavailability of yoga instructors and the difficulties of transportation and travel to large centers, making participation difficult, with large associated costs and time demands (15, 16). Although other remote forms of applying yoga interventions (videos, apps, video conferencing) have been developed and used (17, 18), few studies have investigated how remote Hatha Yoga interventions can be applied in practical real-world situations. Thus, the purpose of this pilot study was to evaluate the feasibility and acceptance of a remote Hatha Yoga intervention (asynchronous videos) and to examine the potential effects on chronic musculoskeletal pain, sleep, and psychological variables (anxiety and depression) of women with chronic pain.

## 2 Method

### 2.1 Design

This is a 2-week feasibility, and controlled pilot study comparing Hatha Yoga with a control group. The study included a sample of women, living in a rural area in southern Brazil, with complaints of chronic musculoskeletal pain. The study was carried out within the ethical standards of the Declaration of Helsinki and following Resolution 196/96 of the Ministry of Health.

### 2.2 Participants

The participants were recruited from a rural region in the city of Itajaí between November and December 2022. The eligibility criteria were: women, over 18 years of age, who had been complaining of musculoskeletal pain for more than 3 months. Women who had practiced yoga in the previous year, or with cognitive or functional limitations that prevented the practice of group physical activity (to minimize the risks of performing an unsupervised exercise intervention) were excluded. Women who had limitations to stand up or lie on the floor (transition required between Hatha Yoga postures) were also excluded.

The recruitment method for the composition of the intervention group and control group included dissemination in the neighborhood community group and messaging groups through mobile applications. Participants who showed interest in participating were invited to an interview in the neighborhood church hall, and those eligible for the study were asked to sign the consent form. Group allocation was determined by a simple random draw.

### 2.3 Intervention and control

**Figure 1:**
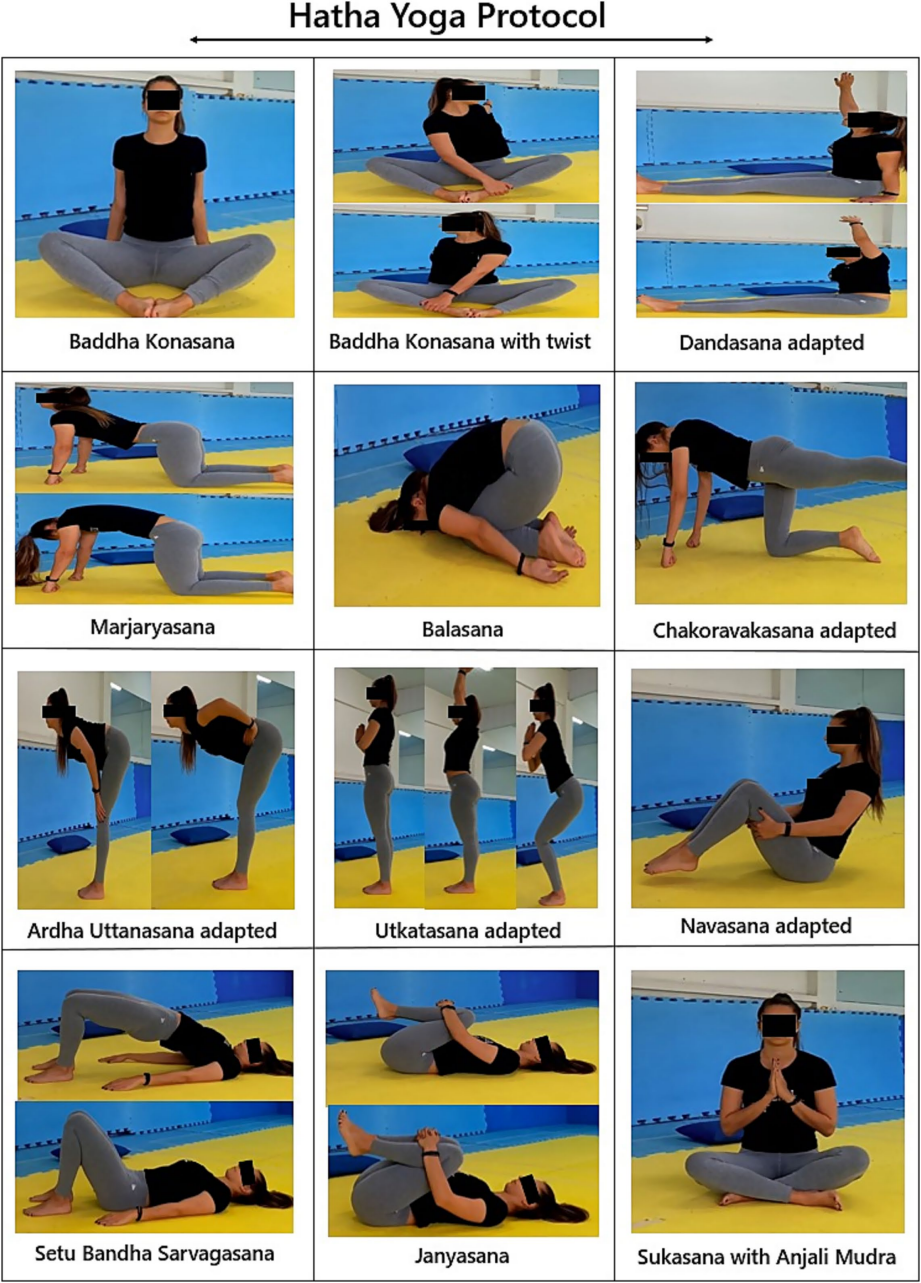
Postures (asanas) used in the Hatha Yoga protocol.

The intervention group participated in video-guided Hatha Yoga sessions (60 min), applied 2 times a week for 2 weeks, in a location provided by the community church. The sessions were accompanied by a physical education professional who started the video and offered instructions, when necessary. The video intervention was specifically adapted to meet the needs of the target population by a certified yoga instructor and included three moments: (1) Stillness and Awareness of Breathing (Pranas and Dayana’s), (2) Practices of the Asana Postures (postures) in the sitting, supine, and standing positions, and (3) and meditation (main postures are illustrated in Figure 1). The sessions were developed to occur fluidly from the beginning, with small transition intervals between postures, facilitating the development of Pratyahara (abstraction of the senses) and command and synchronization of movements with breathing (Table 1 Hatha Yoga Intervention Protocol via video). In addition, rest postures were added, which as well as physical rest, allow moments of recollection and observation of the body and breathing. The video contains instructions on posture variations and the use of a yoga mat, blocks, blankets, and a chair, when necessary, according to the physical limitations of the participants.

**Table 1:** Hatha Yoga exercise program intervention protocol (video).

Division and time in minutes of realization	Practice description
Philosophy from Hatha Yoga (5 min)	Explanations of the importance of the practice and reflections on the philosophy of Hatha Yoga.
Quieting and awareness of the breath (10 min)	Performing breathing exercises (pranas); and Dhyana meditation (meditation) in the positions: Sukusana and lying supine with the knees semi-flexed.
Postures psychophysical (25 min)	Performing ten psychophysical postures (asanas) in the sitting, supine, and standing positions as a way of cultivating the body and mind. The postures worked were: Baddha Konasana, Dandasana, Marjaryasana, Balasana, Tadasana, Chakoravakasana, Uttanasana, Utkatasana and Navasana (Figure 1). Participants were encouraged to stretch as much as possible without exceeding the limits of their comfort. Repetition was consistent week after week and linked pose to pose. Each posture was held for approximately 20 to 30 s, with rest periods lasting 30 s to 1 min between postures. Mats were used to perform the positions. Chairs for. The instruction was to concentrate on breathing and try to relax.
Meditation	Execution of the meditative practice in the positions: Savasana and Sukusana.

The control group participants did not receive any type of intervention and were instructed not to start Hatha Yoga classes or exercise programs during the 2 weeks.

### 2.4 Procedures

**Figure 2:**
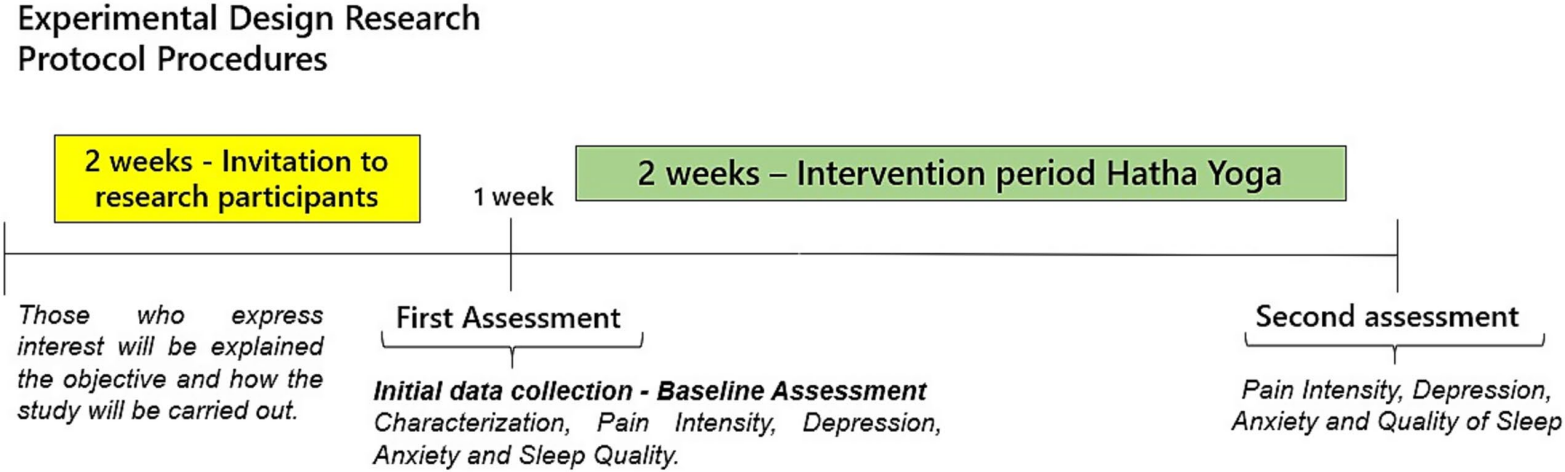
Data collection procedures and steps.

The stages of data collection are described in Figure 2. The outcomes evaluated before and after the intervention were: Pain Intensity, Depression, Anxiety, and Sleep Quality. Feasibility measures were also evaluated: adverse effects/safety, acceptability, ability to complete the intervention, and external interference from other physical activities.

#### 2.4.1 Viability

Safety was assessed through records of adverse events during and outside of yoga classes. Unexpected injuries (falls, fractures, sprains, muscle tension, and/or joint pain) were considered adverse effects. These events were monitored and recorded by the physical education professional who accompanied the sessions.

Acceptability was assessed using a form with questions related to participant satisfaction, that was applied at the end of the intervention. The form consisted of two questions: (1) How do you evaluate the yoga classes? (bad, fair, good, very good, great), and (2) Would you consider continuing to practice yoga? (yes or no).

The ability to complete the intervention was characterized by the ability to perform the postures and movements during the Hatha Yoga intervention and by the need to adapt or modify postures. These aspects were monitored and recorded by the professional present at the sessions.

Contamination was assessed through participants’ reports related to beginning of other exercises during the intervention.

#### 2.4.2 Outcomes

*Pain intensity*—assessed using the Numerical Pain Scale (NPS), a simple scale often utilized to assess variations in subjective pain intensity (19). The scale ranges from 0 to 10, where 0 represents “no pain” and 10 represents “extreme pain.” Participants were asked to state their pain level on the scale at the time of each assessment (before and after the intervention).

*Sleep quality*—assessed using the Pittsburgh Sleep Quality Index (PSQI), a self-report instrument developed by Buysse et al. (20), which assesses sleep quality in the previous month. Our study used a short version of the PSQI that was adapted and validated for Brazil (21). This version assesses sleep quality over the previous 7 days; it consists of 9 questions that assess duration, subjective quality, efficiency, disorders, medication, daytime dysfunctions, latency, and total sleep quality. The PSQI score ranges from 0 to 21; the higher the score, the worse the sleep quality. A score > 5 indicates difficulty in at least two domains.

*Depression and anxiety*—the Beck Depression Inventory (BDI) and the Beck Anxiety Inventory (BAI) were used to assess depression and anxiety levels. Higher scores indicate higher levels of depression and anxiety. Each questionnaire consists of 21 questions, with each question scored from 0 to 3. The BDI scale was validated in Portuguese for Brazil by Gorenstein (22), with a score < 10 being classified as minimal or no depression, 10–18 as mild to moderate depression, 19–29 as moderate to severe depression, and 30–63 as severe depression. A BAI score of 0–21 indicates very low anxiety, 22–35 moderate anxiety, and 36 or more high anxiety.

### 2.5 Statistical analysis

For descriptive analysis, mean, standard deviation, and percentage were calculated. For inferential statistics, normality analysis was first performed with the Shapiro–Wilk test. To analyze the differences between groups, the Wilcoxon test was used, and the Mann–Whitney *U* test was used to compare the control group and the different moments of the intervention group. The *α* established as the significance level was *p* < 0.05 (95%). The data were statistically treated with the Statistical Package for Social Science 20.0® (IBM, USA). The Cohen’s *d* effect size was also calculated; a value of *d* = 0.2 was indicative of a small effect, *d* = 0.5 a medium effect, and *d* = 0.8 a large effect (23).

## 3 Results

### 3.1 Characteristics of the participants

Thirteen women participated in the study, distributed into an intervention group (*n* = 8) and a control group (*n* = 5). The sociodemographic and clinical characteristics at baseline are described in Table 2. The age of the participants ranged from 47 to 77 years (62.9 ± 9.3). The participants were predominantly retired (69.2%), with low education (only 2 participants completed high school), and with a family income of less than or equal to 5 minimum wages. The most commonly reported primary sites of pain by the participants were the lower limbs and spine. Mean pain intensity ranged from 1 to 9, with a mean score of 5.2 (± 2.8). Participants in the Hatha Yoga group tended to have lower mean pain scores than those in the control group. Approximately 50% of the intervention group participants were physical activity practitioners, while none of the women in the control group performed physical activity.

**Table 2:** Sociodemographic and clinical characteristics of 13 women with pain complaints who participated as intervention group during 2 weeks of the remote Yoga intervention and 2 weeks in the control group.

Variable	Control (*n* = 5)	Intervention (*n* = 8)
Age	63.4 ± 8.9	62.6 ± 10.7
Marital status		
Single	0 (0.0%)	1 (12.5%)
Married woman	4 (80.0%)	5 (62.5%)
Widow	1 (20.0%)	2 (25.0%)
Professional status		
Active	1 (20.0%)	3 (37.5%)
Retired	4 (80.0%)	5 (62.5%)
Schooling		
Incomplete elementary education	2 (40.0%)	6 (75.0%)
Complete elementary education	2 (40.0%)	1 (12.5%)
Middle school	1 (20.0%)	1 (12.5%)
Family income (MW)		
1–2	3 (60.0%)	4 (50.0%)
3–5	2 (40.0%)	4 (50.0%)
Pain intensity	4.0 ± 03.0	5.9 ± 02.6
Pain site		
Column	4 (80.0%)	4 (50.0%)
Lower limbs	1 (20.0%)	3 (37.5%)
Upper limbs	0 (0.0%)	1 (12.5%)
BMI		
Normal	2 (40.0%)	3 (37.5%)
Overweight	2 (40.0%)	4 (50.0%)
Obesity	1 (20.0%)	1 (12.5%)
Practice of activity physics		
No	5 (100%)	4 (50.0%)
Yes	0 (0.0%)	4 (50.0%)

Family income was categorized between those who received 1–2 minimum wages (MW) and those who received 3–5 MW; BMI, Body Mass Index.

### 3.2 Feasibility

#### 3.2.1 Recruitment

**Figure 3:**
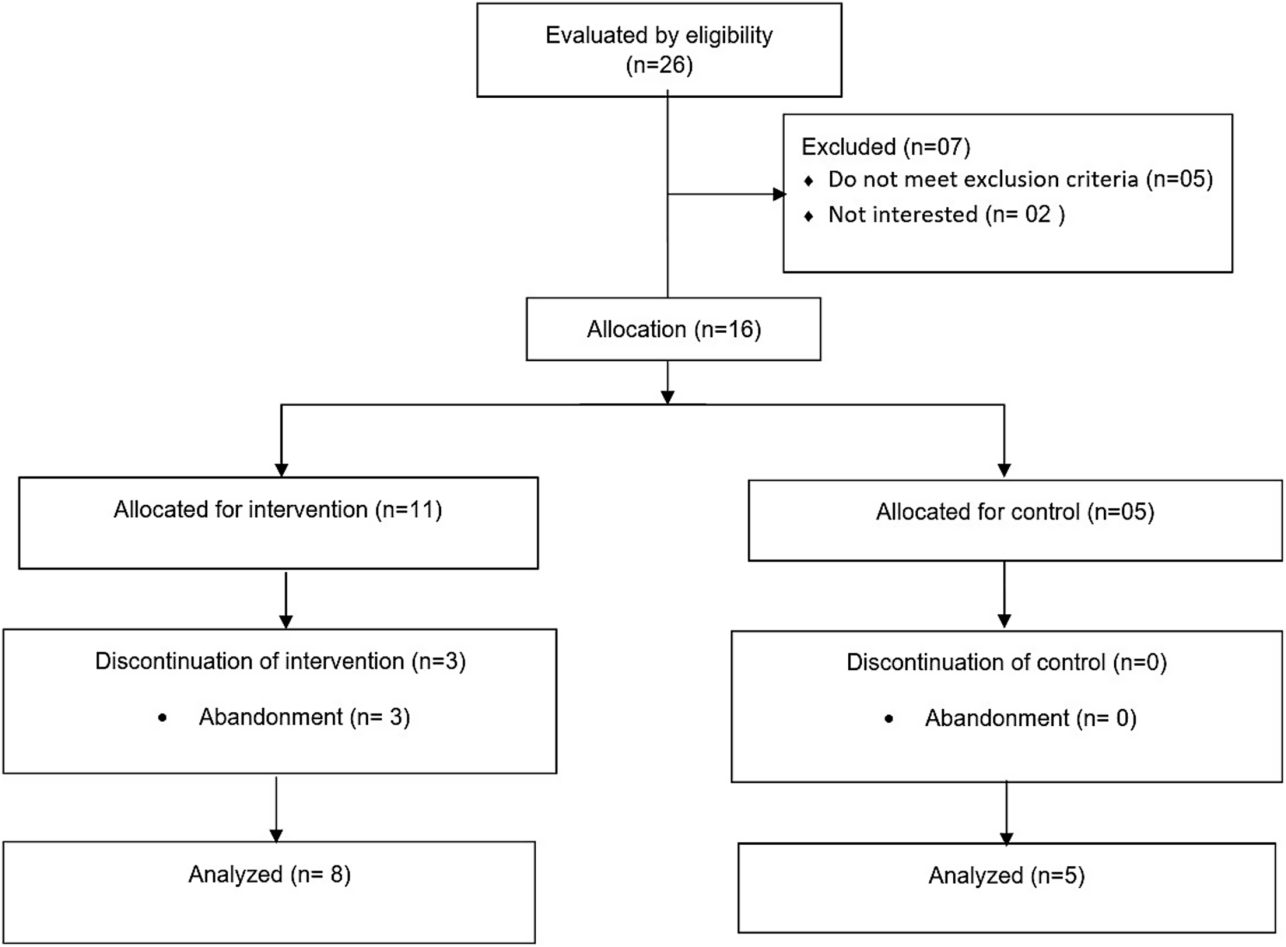
Inclusion–exclusion flowchart of the 26 study participants for eligibility.

A total of 26 women were selected for eligibility, and 13 were deemed eligible for participation. The reasons for ineligibility were: no complaints of pain at the time of the study (03), presented functional limitations (01), and were not available to participate in the study (02). The 16 women considered eligible for participation were divided into two groups: 11 in the intervention group and five in the control group. After the 2 weeks of intervention, there were eight participants in the IG and five in the control group. The reasons for discontinuation in the study were: family commitments (02) and health problems, influenza (01) (Figure 3).

#### 3.2.2 Adverse effects

During the intervention period, only non-serious adverse events (muscle or joint pain) were reported. One participant reported pain in performing the “Adho Mukha Syanasana” posture (Dow Dog Pose) and another reported pain in maintaining the *“Dandayamana Bharmasana”* (Balance Table Pose) posture, as she had reduced flexibility due to retractions resulting from burn scars.

#### 3.2.3 Acceptability

All participants were satisfied with the intervention (25% very good/75% excellent) and would recommend the program to other women (100%).

#### 3.2.4 Ability to complete the intervention

All participants were able to complete the postures and movements presented in the video. The video used for the session suggested alternative postures or adaptations to the postures presented. Two participants asked for verbal guidance to perform the *“Dandayamana Bharmasana”* (Balance Table Pose). No changes to the intervention protocols were necessary after the study began, the participants were able to perform as initially proposed.

#### 3.2.5 Contamination

No participants in either group performed any other physical activity or exercise program during the study period (2 weeks).

### 3.3 Outcomes

In the intragroup comparison, there was a significant improvement in the anxiety status of the intervention group, when comparing pre and post-test, with a *p*-value of less than 0.01 (and an intervention effect size of moderate to large). Although pain intensity did not present significant differences, there was a 1.5 reduction in mean values (moderate effect size). There were no significant differences in the sleep quality of the two groups after 2 weeks of Yoga (post-test) (Table 3). On the other hand, based on the results of the mean values and the effect size presented, there was an improvement in the overall sleep quality on the PSQI and the intervention group depression.

**Table 3:** Effects of two-week yoga intervention on pain, anxiety, depression, and sleep.

Variables	Intervention (*n* = 08)			Cohen’s *d*	Control (*n* = 05)		
	Pre X ± (dp)	Post X ± (dp)	*p*-value		Pre X ± (dp)	Post X ± (dp)	*p*-value
Pain intensity (END)	5.87 ± 2.58	4.37 ± 2.77	0.10	0.50**	4.00 ± 3.00	5.80 ± 1.78	0.102
Anxiety	8.12 ± 6.26	4.12 ± 4.58	0.01*	0.67**	7.60 ± 9.91	5.60 ± 9.78	0.04*
Depression	3.25 ± 2.49	4.12 ± 4.15	0.43	**0.26** ^***^	4.80 ± 7.46	4.80 ± 6.61	1.00
Sleep—PSQI							
Subjective quality	1.12 ± 0.64	1.00 ± 0.92	0.65	0,15	1.20 ± 1.09	1.00 ± 1.24	0.31
Latence	0.87 ± 0.64	1.37 ± 0.91	0.19	**0.42** ^***^	1.20 ± 1.30	1.20 ± 1.30	1.00
Duration	0.87 ± 1.24	0.50 ± 1.06	0.18	**0.43** ^***^	0.60 ± 1.34	0.60 ± 1.34	1.00
Efficiency	1.25 ± 1.28	0.75 ± 1.16	0.19	**0.42** ^***^	0.60 ± 1.34	0.60 ± 1.34	1.00
Disturbance	0.87 ± 0.64	1.12 ± 0.35	0.31	**0.33** ^***^	1.40 ± 0.89	1.40 ± 0.89	1.00
Medication	0.75 ± 1.38	0.37 ± 1.06	0.31	**0.33** ^***^	0.60 ± 1.34	0.00 ± 0.00	0.31
Dysfunction	0.50 ± 0.75	0.37 ± 0.74	0.31	**0.33** ^***^	0.40 ± 0.89	0.40 ± 0.89	1.00
PSQI total score	6.25 ± 4.92	5.12 ± 4.70	0.68	0.14	5.80 ± 7.98	5.20 ± 6.64	0.31

^*^Significant difference *p* < 0.05; Wilcoxon test was used; X, average; (SD), standard deviation; ^**^Effect size ≥ 0.50 is considered a good effect size. ^***^Effect size ≥ 0.20 is considered a small effect size. The significant differences and observed effect sizes are highlighted in bold.

In the intergroup comparison (intervention group and control group) at the post-test moment, there were no significant differences in the variables investigated (Table 4).

**Table 4:** Comparisons between the intervention and control groups.

Variable	Pre-intervention	Pre control	*p*	Post intervention	Post control	*p*
Pain intensity (END)	5.87 ± 2.58	4.00 ± 3.00	0.23	4.37 ± 2.77	5.80 ± 1.78	0.54
Anxiety	8.12 ± 6.26	7.60 ± 9.91	0.55	4.12 ± 4.58	5.60 ± 9.78	0.71
Depression	3.25 ± 2.49	4.80 ± 7.46	0.65	4.12 ± 4.15	4.80 ± 6.61	0.82
Sleep—PSQI						
Subjective quality	1.12 ± 0.64	1.20 ± 1.09	0.86	1.00 ± 0.92	1.00 ± 1.24	0.80
Latence	0.87 ± 0.64	1.20 ± 1.30	0.75	1.37 ± 0.91	1.20 ± 1.30	0.70
Duration	0.87 ± 1.24	0.60 ± 1.34	0.65	0.50 ± 1.06	0.60 ± 1.34	0.92
Efficiency	1.25 ± 1.28	0.60 ± 1.34	0.26	0.75 ± 1.16	0.60 ± 1.34	0.65
Disturbance	0.87 ± 0.64	1.40 ± 0.89	0.28	1.12 ± 0.35	1.40 ± 0.89	0.64
Medication	0.75 ± 1.38	0.60 ± 1.34	0.84	0.37 ± 1.06	0.00 ± 0.00	0.43
Dysfunction	0.50 ± 0.75	0.40 ± 0.89	0.65	0.37 ± 0.74	0.40 ± 0.89	0.92
PSQI total score	6.25 ± 4.92	5.80 ± 7.98	0.40	5.12 ± 4.70	5.20 ± 6.64	0.40

*Significant difference *p* < 0.05; The Mann–Whitney *U* test was used.

**Figure 4:**
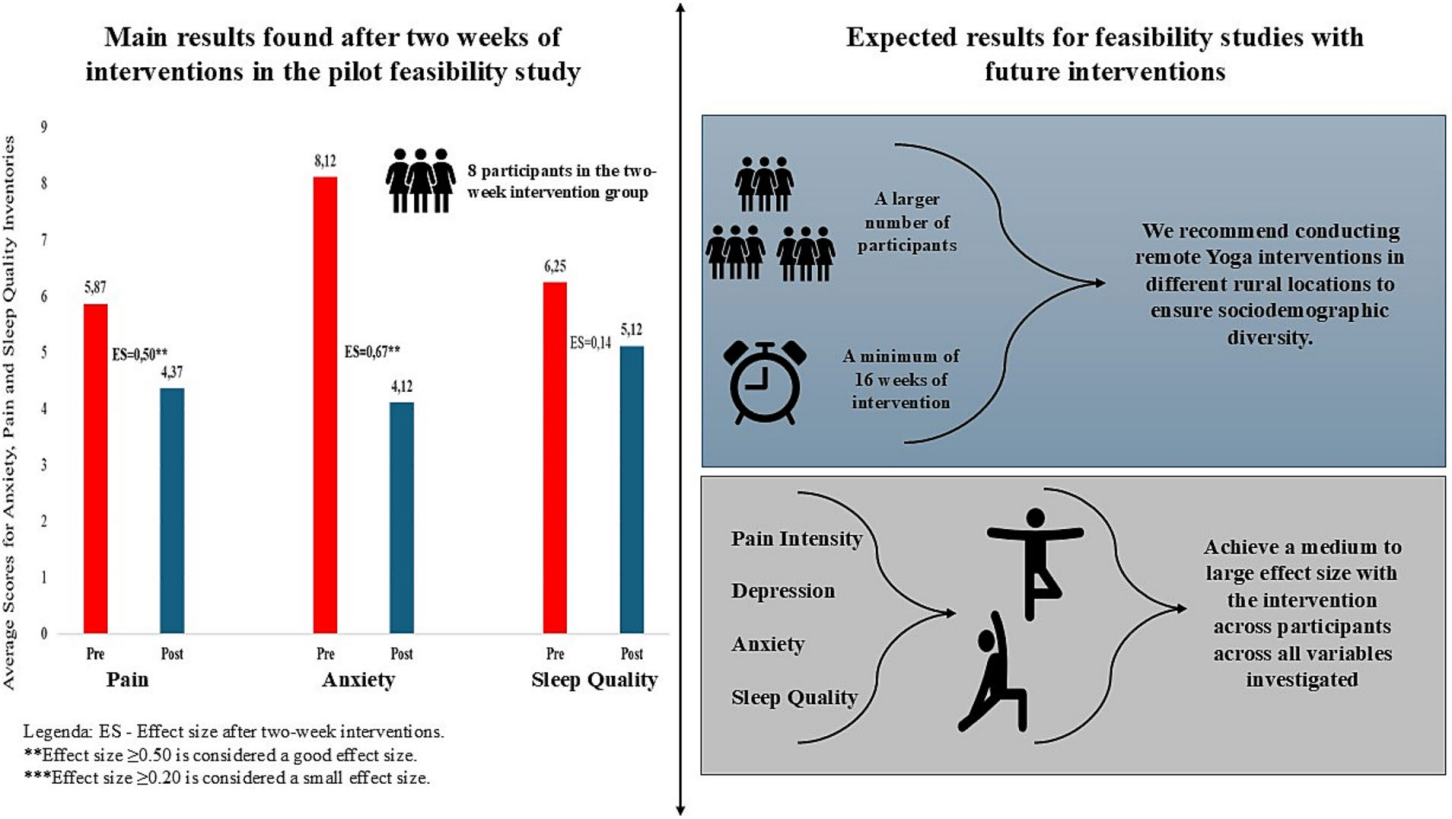
Main results found and expectations for future studies.

Figure 4 presents the main results found and expectations for studies and recommendations.

## 4 Discussion

Chronic pain affects the health and quality of life of older women, limiting their work activities and interfering with social relationships. Several non-pharmacological interventions can contribute to the control of pain and other related symptoms, however, they can be difficult to access for residents of rural areas, as these programs are usually offered in large centers or reference health services. A nationwide systematic review showed that the southern region of Brazil is the most affected by chronic pain in the country (24). Considering the impacts on public health arising from the needs of patients affected by a multitude of diseases, patients being taken to integrated health centers and hospitals due to chronic pain can further inflate the demands on care systems. Thus, studies such as the present feasibility study are needed in more remote locations, such as rural and hard-to-reach areas, as a way to study and mitigate the impacts of pain in various populations that need care.

Over 3 weeks, 16 women were recruited to the study. The research was disseminated by representatives of the community groups, which may have facilitated recruitment, as most of the women already knew each other or had familial ties. The proposal for a Hatha Yoga activity was well received and at the end of the intervention, all participants were satisfied and stated that they would recommend it to other women in the community with chronic musculoskeletal pain. This result indicates the feasibility of carrying out a clinical study using the proposed protocol with a larger number of participants.

No serious adverse events were recorded. As expected, some participants reported pain during some postures and/or the need for adaptations. However, none of the participants failed to carry out the proposed activities. The instructions in the video and the supervision of a trained physical education professional (not specialized in yoga) likely enhanced participants’ confidence and their safety perception. During the 2 weeks of intervention, there was no external interference from other physical activities (none of the participants started another type of exercise), making it possible to evaluate the acute effects of the modality without interference.

### 4.1 Outcomes

In the present study, women with musculoskeletal pain complaints showed a significant improvement in anxiety. This reduction in the BAI score was also seen in the control group, however, the women in the group that practiced Hatha Yoga for 2 weeks presented a greater reduction, with a moderate to large effect size.

There was no significant reduction in pain intensity in either group. The intervention group showed a reduction in the END score after the intervention (moderate effect size), however, of less than two points (or a reduction of approximately 30%), thus not representing a clinically important difference. There were also no significant reductions in the other outcomes, although slight improvements with a small effect size were found in the variables’ depression, latency, duration, efficiency, disorders, medications, and sleep dysfunction. A systematic review with meta-analysis revealed positive effects of yoga in women with sleep problems, using PSQI scores in 16 randomized controlled trials, compared to the control group for the improvement in sleep quality among women (25), which strengthens our results. The lack of improvement in these outcomes, in both sleep and pain, may be due to the small sample size, or point to the need for longer exposure to the intervention to produce a pain reduction and improve sleep quality. Furthermore, psychological constructs like fear of pain have been shown to play a key role in chronic pain experiences (26), supporting the relevance of evaluating cognitive-emotional factors together with physical symptoms.

Considering the current study and the literature investigating the problem, we present recommendations to treat chronic pain through evidence-based yoga interventions (Table 5).

**Table 5:** Evidence-based recommendations for yoga interventions to treat chronic pain.

Yoga interventions (27, 28)
*Weekly frequency*: The weekly frequency of the practice can vary from two to 7 days a week with a duration of 30 min each session. Best effects are demonstrated from daily practice between 20 to 30 min duration for each session. *Duration of practice time*: Programs lasting 4–12 weeks have demonstrated efficacy in reducing pain and improving related symptoms. When performed for a minimum of 12 weeks, the effects can be observed by a follow-up of up to 24 weeks. *Reproducibility*: Hatha yoga interventions can be designed for individuals who have never practiced yoga in order to maximize generalizability, efficacy, and safety in a population with chronic low back pain.
Adaptations of interventions (28)
*Customization of the yoga program for the practice*: Adaptations such as the use of chairs close to the participants to assist in lying down and standing up on tatami mats and mats specific to the practice where yoga movements are usually performed, can reduce barriers both to the positioning of some movements and to bring safety to participants when changing positions. *Adaptations of different movements*: Knowing that each patient has a specific comorbidity, and also how they deal with pain individually, adaptations of some movements are recommended when the patient cannot reach the basics in some specific movements due to their limitations.
Artificial Intelligence in the Application of Yoga (29, 30)
*Machine Learning*: Benefits of pose estimation can be used for yoga to help users assume Yoga postures with better accuracy. The yoga practitioner can detect their current posture in real time, and the pose estimation method can provide corrective feedback if they make mistakes. *Machine Learning and remote Yoga interventions*: Real-time yoga pose estimation can be an advance in remote virtual Yoga instructions by the specialist teacher, facilitating supervision during interventions, becoming an excellent tool, for example, in moments of interventions during major pandemics.
Adherence to practice (28, 31)
*Adherence*: Evidence has shown that underlying pain conditions, the presence of comorbidities, and a lower level of socioeconomic status are the characteristics of participants who most adhere to yoga interventions. *Practice adherence strategies*: Yoga interventions integrated with other complementary medicine therapies can increase pain patients’ adherence to the practice.
Physical and mental benefits (4, 27, 28, 32, 33)
*Online interventions*: Virtual hatha yoga classes may be a viable, safe, and effective treatment option for adults between the ages of 18 and 64 with chronic low back pain. *Comparison of conventional treatments with yoga:* Objective evidence demonstrates that Medical Yoga Therapy relieves chronic low back pain, and stress, and improves quality of life better than standard treatment. *Sense of control*: Yoga has robust short- and long-term effects on pain, disability, physical function, and mental health when compared to control groups without exercise. *Impacts on sleep*: Yoga interventions were beneficial for better sleep quality in patients with chronic pain.
Public Policies (28, 31, 34)
Implementing campaigns and public policies that encourage the regular practice of virtual yoga classes can reduce barriers to face-to-face participation, being a viable, safe, and effective treatment option for patients with chronic low back pain. Studies have shown that the majority of practitioners with chronic pain who seek yoga for pain relief are covered by health insurance, which indicates the need for interventions at the public policy level to increase the use of yoga by the general public, despite its growing popularity.

### 4.2 Limitations and future studies

The current study did not have sufficient statistical power to detect differences between the groups and 2 weeks may have been insufficient to observe significant effects, however, the reduction in anxiety with a moderate to large effect size and the trend to improvement in the other outcomes provide support for larger-scale controlled studies and comparisons between programs of different durations. In our study, the participants had similar characteristics (low education, limited income, older age), which may limit the application of the results to other communities. Therefore, we suggest that future studies be conducted in different rural locations to ensure sociodemographic diversity; and for random group assignment, that sequence generation, allocation concealment, and group implementation be described in greater detail. The Numerical Pain Scale was used as an instrument for the pain outcome due to its ease of application in the context of the study. However, as this is a one-dimensional instrument that only measures pain intensity, it is possible that multidimensional pain instruments or those that assess the impact of pain on functionality could provide more information about the effect of the intervention on this outcome.

### 4.3 Strengths and applications

This study addresses a significant public health problem, chronic pain, with a focus on women in rural communities, a group often overlooked in clinical trials. Our results demonstrate that a remote intervention is possible and well-accepted in a population with limited access to resources. The use of asynchronous videos for yoga practices with supervision by health professionals (without yoga training) makes the intervention replicable in other remote regions with limited resources. The high satisfaction rate of the participants and the absence of serious adverse events are positive results. We reinforce the need for future studies with larger samples, considering different durations of intervention and with active control groups (such as relaxation or stretching interventions, to reduce the impact of the placebo effect) and participants from different rural locations, in order to assess the generalizability of the results.

## 5 Conclusion

An asynchronous Hatha Yoga intervention designed for people with musculoskeletal pain, even in a short period, was feasible and safe to apply to a group of women with chronic pain, residents of a rural community, who had moderate pain. Despite the short duration of the Hatha Yoga intervention, the data presented suggest that improvements in anxiety, pain, depression, and sleep quality were observed in women with chronic pain. Adverse events were mild, and the intervention was well accepted by the participants, with a high satisfaction rate.

## Data Availability

The raw data supporting the conclusions of this article will be made available by the authors without undue reservation.
