# Correction: Immediate effects of yoga on anxiety, depression, and sleep in women with chronic pain in a rural community setting: a pilot feasibility study

**DOI:** 10.3389/fmed.2025.1760969

**Published:** 2025-12-16

**Authors:** Frontiers Production Office

**Affiliations:** Frontiers Media SA, Lausanne, Switzerland

**Keywords:** anxiety, depression, mental health, pain, sleep quality, yoga

An incorrect **Funding** statement was provided. The correct funding statement reads:

The author(s) declared that financial support was received for this work and/or its publication. This work has been supported by the following Brazilian research agencies: Foundation for research and innovation support of the State of Santa Catarina—FAPESC, Research Program for the SUS: Shared Management in Health—PPSUS 16/2020; e PAP 027/2020 by the Coordination for the Improvement of Higher Education Personnel—Brazil (CAPES)—Funding Code 001, and by the National Council for Scientific and Technological Development (CNPq). Coordination for the Improvement of Higher Education Personnel—CAPES Functional Scholarship at Doctoral level—NOTICE PPGCMH N 004/2024; There was also a financing contribution from of the Italian Ministry of University and Research pursuant to D.D. No. 1159 of July 23, 2023 - PROBEN Call.

The original version of this article has been updated.

